# Providers’ perspectives on implementing alert-based patient-reported outcome monitoring for stage IV breast cancer

**DOI:** 10.1186/s41687-026-01106-0

**Published:** 2026-06-03

**Authors:** Clara Breidenbach, Anna Maria Hage, Sophie Klara Schellack, Therese Pross, Adam David Dordevic, Christoph Kowalski, Maria Margarete Karsten

**Affiliations:** 1 Department of Health Services Research, German Cancer Society, Berlin, Germany; 2 Department for Gynecology with Breast Center, Charité – University Medical Center Berlin, corporate member of Freie Universität Berlin and Humboldt-Universität zu Berlin, Berlin, Germany

**Keywords:** Breast neoplasms, Patient-reported outcomes, Patient monitoring, Quality of life, Digital health

## Abstract

**Objectives:**

Using digital patient monitoring with patient-reported outcomes (PROs) is increasing. However, these new digital care tools have not yet been adequately implemented in routine cancer care. To address this issue, we conducted a process evaluation to accompany the PRO B study, a multicenter randomized and controlled trial across Germany, which examined the impact of alert-based PRO monitoring in stage IV breast cancer patients. This article presents the results of this process evaluation from the perspective of participating health-care providers, focusing on determinants for successful implementation.

**Methods:**

Semi-structured interviews with 15 interviewees from 12 certified breast cancer centers across Germany participating in the PRO B study were conducted. Interviews were structured by content analysis using the Consolidated Framework for Implementation Research (CFIR).

**Results:**

Key findings include the pivotal role of study nurses in managing the PRO monitoring system and its alerts, engaging patients, and ensuring continuity of care, alongside the providers’ generally favorable views of the intervention. However, challenges such as limited digital infrastructure and the need for integration into existing hospital information systems were noted.

**Conclusions:**

The study highlights the crucial role of nurses, existing infrastructure, and streamlined processes for integrating PRO monitoring into routine clinical practice. Providers’ experiences with the intervention highlighted the need for workflow adjustments and additional training for effective implementation in routine cancer care. Tailored solutions, including integration into existing digital patient record systems and processes, as well as resource allocation, are essential for the successful adoption of PRO monitoring in a health-care system.

**Supplementary Information:**

The online version contains supplementary material available at 10.1186/s41687-026-01106-0.

## Introduction

Patient-reported outcomes (PROs) can be used for individual patient monitoring to assist patient care and treatment decision-making, as well as for quality improvement initiatives including benchmarking of health-care providers [1]. In the international oncological setting, several studies have already demonstrated the beneficial effects of PRO monitoring, and it is recommended for specific situations in clinical guidelines [2, 3]. The milestone studies by Basch et al. [4] and Denis et al. [5] not only showed significant improvements in quality of life, but also in overall survival in stage IV cancer patients who used alert-based PRO monitoring [6]. 

The two-armed randomized and controlled PRO B study tested whether the approach described in a clinical trial by Basch et al. [4, 7] could be transferred to the German cancer care system, and investigated its effectiveness in a study in 52 study centers across Germany. In this article, we present the results of the process evaluation of the PRO B study from a health-care provider’s perspective. According to the Medical Research Council (MRC) framework — an international widely used and established guidance system in implementation science for developing and evaluating complex interventions — process evaluation of this type is an integral step in the development and implementation of interventions [8]. 

As the PRO B study showed positive effects of the intervention on quality of life and even survival (Karsten et al., submitted), the next goal is to integrate this intervention into routine breast cancer care—something not yet implemented in Germany or most other healthcare systems. An evidence-practice gap is well recognized for PROs, referring to the discrepancy between the evidence supporting practices like alert-based PRO monitoring and their actual implementation [9–11]. As a result, there has been a growing body of research focused on ways of incorporating PROs into routine oncological care to improve health services [12, 13]. While existing studies have identified both obstacles and supportive factors for the implementation of PROs [14, 15], reviews indicate that there has been insufficient focus on the particular contexts in which PROs are applied [14, 16]. 

We therefore carried out a process evaluation of the PRO B intervention with the objective of identifying which aspects may hinder and promote the implementation of the PRO intervention in routine care. The focus was specifically on the German context of stage IV breast cancer, taking both the patients’ and providers’ perspectives into consideration. While the patients’ perspective will be reported separately (Hage et al., submitted), this article reports the results of semi-structured interviews with health-care providers working with the PRO intervention in the PRO B trial. The evaluation was underpinned by the Consolidated Framework for Implementation Research (CFIR). The CFIR is an internationally commonly used data extraction tool for characterizing the determinants of effective implementation of innovations in health care [17]. It contains five main domains: the innovation domain (the “thing” being implemented), the inner setting domain (the setting in which the innovation is implemented), the outer setting domain (the setting in which the inner setting exists), the individuals domain (the roles and characteristics of individuals), and the implementation process domain (the activities and strategies used to implement the innovation). Using a globally recognized analytical framework should facilitate positioning the findings of this research within international literature.

## Methods

### Study design and data collection procedures

#### PRO B study

**Fig. 1:**
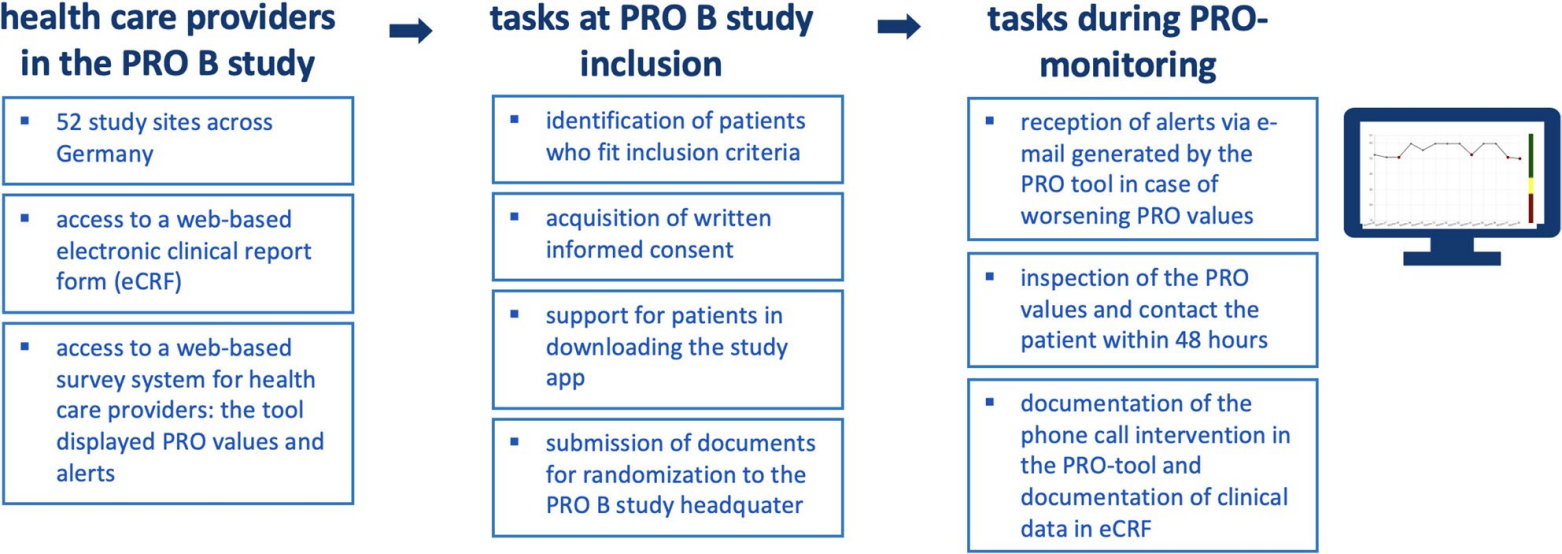
Providers’ study process

PRO B was a multicenter randomized and controlled trial that aimed to investigate the effects of alert-based electronic PRO monitoring in patients with stage IV breast cancer. The primary end point was fatigue at 6 months after randomization, and secondary end points included among others overall survival [6, 18]. From May 2021 to June 2023, 924 patients were enrolled in 52 study centers and the intervention phase took place from May 2021 to March 2024. Patients in the intervention group were electronically assigned a weekly questionnaire with patient-reported outcome measures (PROMs) via a web-based system, and they received them via a mobile application on their smartphones. In case of deteriorating PRO values, the PRO tool generated an alert and sent out an e-mail to breast cancer center treating the patient. Following an alert, the study centers were instructed to contact the patient within 48 h by phone call, and they documented the results of the conversation in a web tool (see Fig. 1 for the providers’ study processes). Patients in the control group received the usual care and were assigned PRO questionnaires every 3 months. Their answers were not connected to an alert system and were not graphically displayed to the care team. All study centers received an expense allowance for each patient enrolled.

#### Process evaluation in the PRO B study

The process evaluation used an exploratory content analysis approach. Data was obtained from semi-structured interviews based on an interview guide with open-ended stimuli and complemented by further narrative-generating questions (see Appendix, Table 1). The interview guide was evaluated by the interviewer after the first interview, but no changes were found to be necessary. Interviews took place during the intervention phase of the [anonymized] study, from May to July 2022. Fourteen interviews with providers were conducted (in one case, two interviewees participated together in one interview). Interviews were conducted by CB (female, PhD candidate and later postdoctoral researcher), who was not part of the study team that designed the original PRO B study and did not act as the contact person for the study centers. She introduced herself to the interview partners in a neutral evaluation role. The interviewer was not known by the interviewees beforehand. The interviews were held via telephone and were audio-recorded. Transcriptions were made by student assistants using the f4transkript program. No repeat interviews were carried out, and transcripts or findings were not returned to participants for comments or correction. A few field notes were made during the interviews, but they were not coded or analyzed. Interviews continued until data saturation was reached.

The Consolidated Criteria for Reporting Qualitative Research (COREQ) checklist was used to report this research. Ethical approval for the PRO Bstudy was obtained from the Charité Ethics Committee (EA2/070/23). All participants provided written informed consent to participate in the study.

## Participant recruitment

The participants were recruited using a snowball technique. In April 2022, we invited 19 of the 52 PRO B study centers with particularly high and low enrolment numbers via e-mail to nominate employees working on the study in their study center to take part in an interview. The providers were eligible for an interview when involved in the PRO B study. Providers who showed interest in being interviewed were contacted again to check eligibility criteria and set up a meeting for the interview.

### Data analysis

The interviews conducted were structured by two coders (CB, SS) using a content analysis approach based on Kuckartz [19]. Initially, a few overall categories were built deductively from the main domains of the CFIR. Then, additional categories and subunits were phrased inductively. Both coders coded each interview independently. They then met to jointly review and discuss any disagreements and find consensus. The final coding tree is presented in the Appendix, Table 2. The software program f4analyse was used to structure the data.

## Results

### Description of the sample

The final sample with data for the providers’ perspective included 15 interviewees from 12 certified breast cancer centers across Germany. Five of the 12 cancer centers were located in university hospitals. The cancer centers ranged in size and were located in urban as well as more rural areas. One male and 14 female providers, with an average age of 42 (23–63), were interviewed. The providers’ sample contained three physicians, 11 study nurses, and one documentation assistant. The interviews lasted between 9 and 31 min (average 17 min and 42 sec) which resulted in averagely 6.4 pages of transcript per interview (font Arial, size 12, minimum 4 and maximum 10 pages).

### Key themes

In the following, the results of the interviews are presented based on the CFIR domains. Within the five domains of the CFIR, we identified topics in three domains: innovation, inner setting and process implementation. To illustrate the themes, we provide examples within the text or refer to quotes (for example, #3) that are listed in detail in the Appendix, Table 2.

#### Innovation domain

The innovation domain includes aspects of the new process or intervention that is being implemented [17]. 

The providers perceived that the PRO B study was an innovation in their routine work and that it differed from other PRO studies they had participated in previously — because in this innovation, the process required them to interact with PRO results when they received an alert, in contrast to other studies in which PROs were simply collected (#1). The providers perceived the alert-based PRO monitoring introduced through the PRO B study as being generally reasonable. They saw the potential of the innovation for supporting patients and the relevance of research on PROs (#2, #3). However, they also described limitations — for example, that they thought PRO questionnaires only reflect the patient’s status at one specific moment and that a conversation with a physician can produce a more holistic picture (#4). It was also mentioned by one provider that she did not perceive any actual practical advantage of measuring quality of life (#5):

That’s why, hmm, so I’m in two minds here a bit, there are studies that do show that quality of life is something extra-important and where with all this management you can produce a clear improvement, but at the moment I don’t think it’s that serious for us yet. (#5, ID15, Physician)

With regard to the perceived advantages of the innovation, the providers described experiences similar to those of the patients in the PRO B study (Hage et al., submitted) — they recognized that patients appreciated the calls and felt secure and in good hands during the intervention (#6):

It’s well accepted. So I think they’re quite pleased if someone sort of asks them again in addition how they’re feeling and they usually always all carry it out. I’ve only heard positive reactions so far. (#6, ID8, Physician)

They also reported that communications between providers and patients were enhanced by the phone calls following an alert. Study nurses were perceived as additional contact persons (#8), and patients even called proactively because they had a principal contact person available in the center thanks to the study (#7):

And you also have an additional face again as well, so as I now always go over to the chemo, you were also an extra contact, because the doctors are always very short of time, the study assistant’s a bit more flexible there. (#8, ID12, Study nurse)

Providers felt that most of the time, phone calls with the patients had a counseling/advisory character and revolved around emotional and psychological support (#9, #10), providing medical information or coping with side effects (#11, #12, #13).

Um, advice rather, so in my case there was never any drug treatment that had to be started, um, it once happened that someone needed to, I think it was about lymphedema and that sort of thing, just with advice about what options there are for reducing pain, one of the doctors actually took that over, but also in the hospital the patient was then actually in the hospital for a routine appointment later the same day, but I’d already informed the doctor before that what to expect and then advice was given, but actually never a drug intervention so far. (#12, ID13, Study nurse)

They felt that clinical actions involving adjusting the treatment due to alerts were rare (#14, #15). However, they reported that the alerts were also helpful in preparing themselves for upcoming consultations with a patient — e.g., for administrative processes in health care such as referral for rehabilitation services or initiating contact with the interdisciplinary team in care (#16, #17).

Exactly, exactly, and [the doctor] was able to get information beforehand, it was about applying for rehab and that kind of thing, how she could help out with that and we were able to solve it the same day, that was by chance that she happened to have an appointment, exactly and then the doctor already knew about it and then it worked out. (#16, ID13, Study nurse)

Then if it’s really serious the doctor informs the psychologist or psycho-oncologist right away, and when I see her I phone up and say I’ve got Ms So-and-So here, but because they’re also linked to us, they very often say yes, I already have an appointment with Ms DotDotDot at such and such a time. (#17, ID14, Study Nurse)

In general, providers perceived that they only had to expend little effort regarding this innovation in comparison with other studies (#20, #21). One provider reported different experience, where they reduced enrollment because they felt stressed by the number of alerts (#22). With regard to the handling of the study, the interviewees addressed two topics: Firstly, they reported some general technical issues such as insufficient Internet connection in some areas of the hospital, which led to problems in downloading and installing the PRO B application in some cases (#18). Secondly, they broached the issues with using the web-based PRO tool that displayed the alerts — they mentioned that it was not possible to immediately recognize which domains of the PRO questionnaire had triggered an alert (#19):

And also you can’t see at first glance what it is that has caused the worsening. At least I can’t. (#19, ID4ID5, Study nurse)

#### Inner setting domain

The Inner Setting Domain describes interactions and changes that implementing the innovation causes in the immediate setting [17]. 

Providers described two main processes within the study that had to be incorporated into routine operating processes: including patients in the study and reacting to alerts. Study inclusion was organized differently from center to center. In addition to physicians including patients during consultations (#23), several structured processes were described — for example, discussing patients’ eligibility for the study in the tumor conference (#24) or having study nurses leave comments on the patient record on whether patients were eligible to remind physicians (#25). The alerts were reported to be handled by study nurses in most cases (#26, #27, #28). They called the patients by phone, discussed their issues, and if needed then consulted or called in a physician:

With issuing receipts for this alarm in the online system, it’s the study nurse who does that with us, but we also had to do it once, once when the study center wasn’t staffed for sick leave reasons, but issuing receipts for alarms is routinely done by the study nurses, clicking on it so to speak. (#27, ID8, Physician)

And then we let the patients describe it to us, then we discuss it with the doctor and depending on what comes out then, the patient is then given an appointment or sent to the family physician, or yes and then we document that in the patient’s course sheet here. (#28, ID4ID5, Study Nurse)

In one center, it was reported that study nurses and physicians shared the answering of the incoming alerts, including the phone call interventions (#29).

#### Process implementation domain

The process implementation domain includes activities and strategies that are used to implement the innovation [17]. 

Providers described the support they received from the PRO B study headquarters during the innovation as being very useful and important (#30, #32). Study initiation was mostly performed using the snowball principle in the study centers: a few staff members took part in the official initiation of the study by the PRO B study headquarters. They then shared the information with the rest of the staff in their center (#30, #31). With regard to implementation of the innovation in routine work outside of a study, providers named several steps that would be required in order to ensure low-threshold use of the innovation (#33): integrating the PRO tool and its alert system into existing hospital information systems (#34) or patients’ records, as well as providing the additional time resources that are needed to monitor patients and call them (#35, #36).

What the handling looks like, so you can implement something like that in everyday clinical work, i.e. from both sides. So by the ones, I don’t even know who you would call a user, whether it’s the ones who read the data out or the ones who put it in. But so both sides can have a process that’s as smooth as possible. (#33, ID1, Physician)

Yes, I do think it’s feasible, just using tablets that are issued when the patients arrive, where they can click on their side effects and that’s fed into the corresponding medical documentation and hospital documentation system and you can also take a look at it and then things develop here so that it becomes usable in routine work, in just the same way as you can enter the patient history and things like that. (#34, ID1, Physician)

So in routine clinical care the shortage of time is already a problem for staff and I think that could be the only obstacle. (#35, ID13, Study Nurse)

So I think it will be very very difficult, because A you already don’t have enough nursing staff and nurses, B they’d have to have special training I think and C I don’t know what things look like for the doctors, they would also have to have the time to check their e-mails every time and our doctors don’t always have time to check all the e-mails, that’s why it’s good that we have enough time, we sort of only work in the office as study nurses to process the alarms. I don’t think there’s really enough time for that in normal routine nursing, because when I look, of course there are probably sisters who like to say OK, I’ll take care of that, but you haven’t got anybody at all at the basic level to see to the patient and it’s a surgical unit. There are often really surgery days from Monday to Friday, but I see that really as a very very difficult point. (#36, ID14 Study Nurse)

## Discussion

In summary, the providers participating in the PRO B study perceived the intervention and its processes and effects as being generally favorable. In addition to the relatively low perceived effort compared to other studies, providers noted positive effects on communication with patients, such as using information from the phone call interventions to prepare consultations. They also observed that patients appreciated receiving phone calls triggered by alerts, valuing the extra attention this provided. This is in line with previous international literature reports in which providers expressed positive views on electronic PRO monitoring and evaluated it as being useful for patient care [20–22]. In the present study, however, in spite of their quite positive direct experience with the PRO B intervention, the providers partly expressed uncertainty and little confidence in dealing with PROs in general. A lack of knowledge about the interpretation of PRO values and handling of PROs has also been reported throughout the international literature on PRO implementation [23]. In a study by Huberts et al., [24] none of the health-care professionals who were interviewed also reported that they received sufficient training on the use of PROs for consultations with patients. PROs and electronic PRO monitoring systems in Germany are still at an experimental stage and are not part of the curriculum in medical schools or the training of nurses. Medical professionals therefore need additional training to understand, interpret, and use PROs in order to cooperate with the implementation of PRO monitoring in routine care. This view is also supported by others such as Huberts et al., [24] who emphasize the need for training at different levels of education, and Stover et al., [25] who go even further by highlighting that stakeholder engagement, in this case of health-care professionals, is necessary in all phases of the development of a PRO intervention and critical for its successful implementation.

The results of this study provide insights for process optimization when considering the implementation of alert-based PRO monitoring in routine care in Germany. To begin with, the interviews demonstrate that study nurses hold a crucial gatekeeping role in the PRO B intervention — they are described as undertaking essential tasks within the intervention such as identifying eligible patients, supporting patients with downloading and installing the application, and last but not least coordinating alerts, carrying out the phone call intervention, and deciding whether a physician needs to be consulted. In interviews with patients in the PRO B study (Hage et al., submitted), it becomes clear that one of the effects of the intervention — conveying a feeling of security and being in good hands — is largely influenced by the communication skills and empathy of the study nurses. However, in the same way as in the user perception analysis of the PRO-TECT trial by Basch et al., [20] providers in the PRO B study felt that incoming alerts in routine patient care outside of a trial setting were a possible burden of on top of their existing workloads. Fundamental adjustments to workflows and staffing are therefore essential to allow electronic PRO monitoring systems to be fully adopted by providers [20, 24]. These changes are needed in order to allocate responsibilities, define roles for PRO monitoring, and for managing of incoming alerts in order to prevent providers from feeling overwhelmed by an intervention that actually enhances their ability and possibly efficiency in managing patients effectively. Huberts et al. [24] point out the need for a process that reduces the burden on health-care providers for successful implementation in routine care. Furthermore, the health-care providers included in this study noted that for implementation in routine care, it is crucial to integrate electronic PRO systems into already existing electronic health records and documentation systems to ensure a good user experience, as has been reported previously [20, 21, 26]. There need to be low thresholds for accessing the PRO data and smooth workflows to increase acceptance of the intervention. This finding is in accordance with other international literature reports from studies that investigated barriers and facilitators for the implementation of electronic PRO systems [21, 27]. As already implied in the introduction, digitalization in the German health-care sector is not as advanced as in other countries and faces various challenges — strict data protection requirements regarding medical data and the lack of digitalized health records require tailored solutions for the German setting, such as strict consent requirements, which increase administrative effort and have to be considered in staffing models.

Having identified these specific characteristics of the PRO Bintervention in the specific context of breast cancer centers in Germany, a review of the existing literature may provide initial approaches to ways of implementing the PRO B intervention in routine cancer care. With study nurses being identified as crucial gatekeepers in the PRO B intervention and context, the recommendations developed for PROs in nurse-led services by Drury and colleagues [28] with regard to providing specialized PRO training, integrating PROs into data flow processes, as well as clear workflow protocols and resource allocation may be of special relevance. Substantial leadership has also been identified as a factor for boosting motivation among providers to integrate PROs [29]. Ernst et al. [30] also provide an implementation framework that makes it possible to evaluate country-specific health-care systems and generate lessons for regional, state-level, and national PRO initiatives. The interviews revealed that each center had to independently decide how to integrate the new PRO processes into their existing workflows. Centers reported, for example, that study nurses discussed patients’ eligibility for the study during tumor board meetings or left notes in patient records to remind physicians about eligibility. It is therefore essential that each center identifies a process that fits its specific workflows and organizational structures. At the beginning of the PRO B trial, training sessions were offered to all participating centers. Some centers reported that only selected individuals attended these sessions and subsequently passed on the information to the rest of the staff. Future training programs could include examples of best practices and encourage internal meetings within the centers to discuss how PROs can be effectively integrated into their unique processes and infrastructures. Additionally, interviewees highlighted the necessity of resources to ensure the PROs can be maintained for long-term use. Many global studies have identified insufficient resources as a significant obstacle [31]. For example, France’s national reimbursement model has facilitated the broad adoption of electronic PRO monitoring [32]. 

Two major limitations have to be considered when interpreting the results of the qualitative exploratory results presented here. Firstly, no providers from centers that failed to enroll patients at all in the PRO B study were interviewed. Most interviews were held with staff from centers that had high enrollment rates in the PRO B study. This means that the results presented lack the point of view of centers that were not able or were unwilling to provide the intervention. During the course of the study, reasons reported by the centers for not participating in the study included: challenges due to the COVID-19 pandemic, a lack of resources, or expecting a workload that was too high. In the future, these perceived obstacles could be addressed by strengthening communication between centers, allowing them to share their experiences with PROs and their strategies for integration. Moreover, it can reasonably be assumed that willingness to participate in the interviews was more likely among highly motivated staff, and this might also bias the results. Regarding generalizability, it should also be noted that all centers participating in the trial were centers certified by the German Cancer Society. Therefore, the centers already had to provide certain structures before the trial, e.g. a study infrastructure. Findings may not apply to providers that do not have these structures in advance. It is also notable that there are large differences between interviews’ duration which was due to the individual amount and manner of talking by the interviewees (speech rate, talking concisely). Moreover, we were only able to interview one male provider which might bias the results – however, it also reflects the reality since the majority of study nurses is female in Germany. Finally, due to the interviewed staff’s limited time resources transcripts were not returned to the interviewees for comment and correction.

## Conclusions

This study provides insights on process optimization when considering the implementation of alert-based PRO monitoring in routine care for stage IV breast cancer patients in Germany. Interviews with providers show that study nurses hold a crucial gatekeeping role in the intervention, and also that providers’ attitudes toward and experience with the intervention described are favorable, while general knowledge about PROs is quite low. For successful implementation of the intervention in routine breast cancer care in Germany, the PRO monitoring needs to be integrated into existing workflows in such a way that it is not perceived as an additional workload and is integrated seamlessly into existing digital health record systems within breast cancer centers.

## Supplementary Information

Below is the link to the electronic supplementary material.

Supplementary Material 1

Supplementary Material 2

## Data Availability

The datasets generated and/or analysed during the current study are not publicly available due to data protection laws.

## Abbreviations

COREQ checklist: Consolidated Criteria for Reporting Qualitative Research checklist
CFIR: Consolidated Framework for Implementation Research
MRC framework: Medical Research Council framework
PROs: Patient-reported outcomes
PROMs: Patient-reported outcome measures
