# Supplementary material for: Providers’ perspectives on implementing alert-based patient-reported outcome monitoring for stage IV breast cancer
Source: J Patient Rep Outcomes. 2026 Jun 3;10:96. doi: 10.1186/s41687-026-01106-0 (PMC13241327; doi:10.1186/s41687-026-01106-0)
Supplement: Supplementary file 1 — Supplementary Material 1 [file 41687_2026_1106_MOESM1_ESM.docx]

**Appendix table 1. Interview guide providers**

| Main questions | Additional questions |
| --- | --- |
| 1. To begin with, a few brief details about yourself: | - Date of birth - -Position/professional experience - -Specialist area - -Experience with PRO B |
| 2. did you already have experience with Patient-Reported Outcomes before the PRO B study? | - What do you think of Patient-Reported Outcomes? |
| 3. how does the PRO B study work for you?  - General narrative | To what extent...   - are there hurdles and problems with the PRO B trial? - do the processes of the PRO B trial influence your work or the work of other practitioners? - Do the processes and work steps of PRO B fit in with existing work steps? - Does the PRO B study influence the distribution of resources, labour and responsibilities in the team? - Do co-workers need to be trained to carry out the PRO B study? - Does PRO B have an influence on conversations/interaction with patients? |
| 4. What do you think of the PRO B study? | In your opinion, what are the advantages and disadvantages of the PRO B trial?  To what extent do patients recognise the purpose/benefits of the PRO B Study?  To what extent do other employees/your centre recognise the purpose/benefits of the PRO B Study?  How challenging is it to describe the PRO B trial, e.g. when providing information? |
| 5. which aspects and processes are working well?  Which processes and aspects do you think should be adapted? | - Patient Concept App for patients - Patient Concept centre area - Alarm acknowledgement - PRO B Doc - Procedure - Training (initiation videos) - - Communication |
| 6. To what extent can you imagine using the procedure in the PRO B study in routine practice? |  |
