# Supplementary material for: Providers’ perspectives on implementing alert-based patient-reported outcome monitoring for stage IV breast cancer
Source: J Patient Rep Outcomes. 2026 Jun 3;10:96. doi: 10.1186/s41687-026-01106-0 (PMC13241327; doi:10.1186/s41687-026-01106-0)
Supplement: Supplementary file 2 — Supplementary Material 2 [file 41687_2026_1106_MOESM2_ESM.docx]

Appendix, Table 2. Coding tree.

| **CFIR construct** | Definition | Example providers |
| --- | --- | --- |
| **Innovation characteristics: The “thing” being implemented.** | | |
| Relative advantage/ Perception of utility | Appraisal whether the innovation is better than other available innovations or current practice. | *Subcategory “Distinction from other interventions/routine”:*  #1: “Em, yes, but not really for very long yet. There’s a study in the prostate cancer center, mainly about this patient-reported outcome sheet and exactly yes, about quality of life for the patients. But otherwise they’re always regarded as a kind of also-ran, more or less as an extra project on the side, but never so exclusively that you would really have sort of directly examined something or observed something for.” (ID10, para. 9)  *Subcategory “Reasonableness”:*  #2: “ […] Doctor X supervises all the studies, she’s our main principal investigator and, em, I know she also thinks the study’s good, because she says the same, that some patients just need to have support with something like that, with these questionnaires and us speaking to them […].” (ID9, study nurse, para. 65)  #3: “I think that’s definitely a point that you would need to sort of go into in more detail. That’s not completely new and has been investigated for several years already, but also absolutely has to be continued.” (ID1, doctor, para. 8)  #4: “[…] where I think with these EORTC sheets, if you give them to the patients they do get marked with ticks somehow, but then when talking to the doctor he says he’s had completely different symptoms in the meantime. So I just think, during a study these sheets at a specific time point always only record the current state, at the moment when he’s filling the questionnaire out […]” (ID14, study nurse, para. 11)  #5: “That’s why, hmm, so I’m in two minds here a bit, there are studies that do show that quality of life is something extra-important and where with all this management you can produce a clear improvement, but at the moment I don’t think it’s that serious for us yet.” (ID15, doctor, para. 23)  *Subcategory “Feeling of security/ being in good hands”:*  #6: “It’s well accepted. So I think they’re quite pleased if someone sort of asks them again in addition how they’re feeling and they usually always all carry it out. I’ve only heard positive reactions so far.” (ID8, doctor, para. 33)  *Subcategory “Facilitating communication with providers”:*  #7: “And it’s also happened a few times that the patients also phone in without there being an alarm. For example, if they’ve got a question at the moment or want to have something explained independently of Pro B, because they’re in the study and they’ve got a direct contact person.” (ID4ID5, study nurses, para. 25)  *Subcategory “Reacting to symptoms/alarms”:*  #8: “And you also have an additional face again as well, so as I now always go over to the chemo, you were also an extra contact, because the doctors are always very short of time, the study assistant’s a bit more flexible there.” (ID12, study nurse, para. 71)  #9: “Hmm, yes, we’ve already had that twice, and twice it was, em, how can I put it, so she didn’t really have health problems, it was more, it was more sort of psycho-oncological and then we were able to clear it up, talk about it.” (ID10, study nurse, para. 33)  #10: “With her I sensed, okay there isn’t really a health problem now, she’s not unwell, but rather she’s just sort of having an emotional low point, em, so we were able to talk about that, then we offer psycho-oncological support and…” (ID10, study nurse, para. 35)  #11: “Hm, so I think that’s happened with one or two patients now, that we really had to do something due to diarrhea or nausea. Otherwise it’s usually things that we can clear up or I can clear up with her alone. Em, so things that aren’t anything medically important, nothing to do with medication, but where the patient really wants to tell me on the phone herself what was behind her change for the worse.” (ID12, study nurse, para. 27)  #12: “Um, advice rather, so in my case there was never any drug treatment that had to be started, um, it once happened that someone needed to, I think it was about lymphedema and that sort of thing, just with advice about what options there are for reducing pain, one of the doctors actually took that over, but also in the hospital the patient was then actually in the hospital for a routine appointment later the same day, but I’d already informed the doctor before that what to expect and then advice was given, but actually never a drug intervention so far.” (ID13, study nurse, para. 19)  #13: “No, so based on the alarms we’ve found there that you do really have to adjust the treatment, so it wasn’t any side effects of chemotherapies, but rather let’s say because the patient had a case of flu, she took Grippostad as well, so over-the-counter agents, or with one patient just because she had more severe nausea, but she’d already had that in the chemo room beforehand, she got a higher dosage of anti-nausea drugs for example.” (ID14, study nurse, para. 21)  #14: “Exactly, so it’s fairly rare that we have an urgent need to do something.” (ID13, study nurse, para. 11)  #15: “Yes, yes. Well, so far it’s really always that we’ve never had to do anything yet. So it all really just sorted itself out, most of them don’t really want to talk to a doctor, they always say they’ll wait till the next appointment and most know all the symptoms already, it’s not dramatic or new in any way, I’d say, what has now suddenly come up somehow. And yeah, there were two already in hospital, so it was already taken care of like that.” (ID6, study nurse, para. 23)  #16: “Exactly, exactly, and [the doctor] was able to get information beforehand, it was about applying for rehab and that kind of thing, how she could help out with that and we were able to solve it the same day, that was by chance that she happened to have an appointment, exactly and then the doctor already knew about it and then it worked out.” (ID13, study nurse, para. 21)  #17: “Then if it’s really serious the doctor informs the psychologist or psycho-oncologist right away, and when I see her I phone up and say I’ve got Ms So-and-So here, but because they’re also linked to us, they very often say yes, I already have an appointment with Ms DotDotDot at such and such a time.” (ID14, study nurse, para. 19) |
| Complexity | This section revolves around whether the innovation is complicated, which may be reflected by its scope and/or the nature and number of connections and steps. | *Subcategory „General technical issues”:*  #18: “For example we’ve got WLAN here in the hospital, which we can share or make available. The patients then have problems with reception or with the data volume, so it’s that kind of trifle that just comes up in the daily routine.” (ID2, study nurse, para. 40)  *Subcategory “Confirmation of alarms”:*  #19: “And also you can’t see at first glance what it is that has caused the worsening. At least I can’t.” (ID4ID5, study nurse, para. 31) |
| Effort | This section revolves around the perceived amount of time and work the study causes. | #20: “[…] extremely low-threshold. It doesn’t really cause me any work at all.” (ID1 doctor, para. 10)  #21: “Not really, you know, but rather a less elaborate study.” (ID4ID5, study nurses, para. 62)  #22: “Also it is actually the case that we reduced our recruitment numbers when we noticed it won’t work like that or we’re getting stressed out a bit with the phone calls. Sometimes we had several per week and then we first reduced it a bit and now we’re going to take it up again. So that we just bring a few more patients in again, we haven’t got that many yet.” (ID15, doctor, para. 23) |
| **Inner Setting Domain: The setting in which the innovation is implemented.** | | |
| Integration in patients’ lives/ in daily processes of involved staff |  | *Subcategory “Study inclusion”:*  #23: “So our patients come to the oncology consulting hours, where the doctors pay attention to whether the patients will be suitable for the study or not. The doctors then speak to them of course, give them information and ask if they’d be interested. And then we, the doctors, hand out a consent declaration form that is signed and returned to us. And then of course the patients are registered, documented in the program.” (ID2 study nurse, para. 10)  #24: “So we’ve got a special field, during registration for the tumor conference, whether they’re suitable for a study or not and then we can, we have the option of choosing PRO B or the other studies, and that’s decided on accordingly. So when we write it in there, the doctors usually have nothing against it, let’s say, exactly, and then we take care of everything to do with it, so that the informed consent discussion is arranged.” (ID6, study nurse, para. 9)  #25: “We’ve designed ‘To Do’ notes that we attach to the files so the doctors know this is a patient who’d be suitable for the study.” (ID4ID5, study nurses, para. 75)  *Subcategory “Alters”:*  #26: “Exactly, so far I’ve always been able to clear it up myself with the patients. To start with I probably also asked the doctors, of course, whether they wanted to make contact themselves but then they always said, you’ve got so much experience, you know most of the patients, if there’s anything there that looks wrong or you think someone needs to do something, then feel free to give us feedback, but as far as I’m able to clear it up alone, then it was also okay.” (ID11, study nurse, para. 23)  #27: “With issuing receipts for this alarm in the online system, it’s the study nurse who does that with us, but we also had to do it once, once when the study center wasn’t staffed for sick leave reasons, but issuing receipts for alarms is routinely done by the study nurses, clicking on it so to speak.” (ID8, doctor, para. 37)  #28: “And then we let the patients describe it to us, then we discuss it with the doctor and depending on what comes out then, the patient is then given an appointment or sent to the family physician, or yes and then we document that in the patient’s course sheet here.” (ID4ID5, study nurse, para. 13)  #29: “We’ve got a kind of call-back list or a to do list here that’s taken along all day through the consulting hours and where people are constantly clicking on it to include some phone calls or call-backs and anyone working with us here who has a second to spare picks up something or other from the To Do list and calls the patients back, of course they’re prioritized and then it’s also run like that. It just gets clicked into the list by the study administration office or the doctors, somebody looks to see which patient it is and then we call her up.” (ID15, doctor, para. 37) |
| **Implementation Process Domain: The activities and strategies used to implement the innovation.** | | |
| Engagement | This section revolves around how participation in the innovation was attracted and encouraged. | #30: “So when the study came to us we discussed that in the team, how it should be managed and when there are questions you can always check with the study administration office if needed. If any technical problems or that kind of thing were to come up, but it was discussed once in the team right at the start how it should sort of run.” (ID8, doctor, para. 29)  #31: “Em yes, we’ve taken part, we I think we’ve taken part several times, I’m not so sure now exactly how many. But we always discuss it at our team session, we have that once a month, it’s always pointed out that the PRO B study is there and that we can recruit for it and — we always have a changing team a bit, the doctors rotate and people who are new to working in outpatient chemo also then start working in studies and of course they’re trained and given information about that.” (ID9, study nurse, para. 71) |
| Support during intervention | This section revolves around how support during intervention was perceived. | #32: “No, it really leaves nothing to be desired. You always have, somebody’s always there who can help you and if you get the wrong contact person or they happen not to know, then you’re passed on really quickly, it’s really great.” (ID7, study nurse, para. 39) |
| Implementation into routine practice | This section revolves around aspects that refer to the integration of the study’s concept into clinical routine care. | #33: “What the handling looks like, so you can implement something like that in everyday clinical work, i.e. from both sides. So by the ones, I don’t even know who you would call a user, whether it’s the ones who read the data out or the ones who put it in. But so both sides can have a process that’s as smooth as possible.” (ID1, doctor, para. 28)  #34: “Yes, I do think it’s feasible, just using tablets that are issued when the patients arrive, where they can click on their side effects and that’s fed into the corresponding medical documentation and hospital documentation system and you can also take a look at it and then things develop here so that it becomes usable in routine work, in just the same way as you can enter the patient history and things like that.” (ID1, doctor, para. 47)  #35: “So in routine clinical care the shortage of time is already a problem for staff and I think that could be the only obstacle.” (ID13,study nurse, para. 49)  #36: “So I think it will be very very difficult, because A you already don’t have enough nursing staff and nurses, B they’d have to have special training I think and C I don’t know what things look like for the doctors, they would also have to have the time to check their e-mails every time and our doctors don’t always have time to check all the e-mails, that’s why it’s good that we have enough time, we sort of only work in the office as study nurses to process the alarms. I don’t think there’s really enough time for that in normal routine nursing, because when I look, of course there are probably sisters who like to say OK, I’ll take care of that, but you haven’t got anybody at all at the basic level to see to the patient and it’s a surgical unit. There are often really surgery days from Monday to Friday, but I see that really as a very very difficult point.” (ID14 study nurse, para. 55) |
